# Evaluation of Efficacy of Cannabis Use in Patients With Attention Deficit Hyperactivity Disorder: A Systematic Review

**DOI:** 10.7759/cureus.40969

**Published:** 2023-06-26

**Authors:** Divyanshu Dhamija, Adedamola O Bello, Asma A Khan, Sai Dheeraj Gutlapalli, Mehvish Sohail, Priyansh A Patel, Sidharth Midha, Surmai Shukla, Lubna Mohammed

**Affiliations:** 1 Internal Medicine, California Institute of Behavioral Neurosciences & Psychology, Fairfield, USA; 2 Psychiatry, St. Martinus University, Pontiac, USA; 3 Internal Medicine, Richmond University Medical Center, Staten Island, USA; 4 Internal Medicine, Medical College Baroda, Vadodara, IND; 5 Radiology, Bharati Vidyapeeth, Pune, IND; 6 Internal Medicine, Qingdao University College of Medical Science, Qingdao, CHN

**Keywords:** adhd, attention deficit disorder with hyperactivity, attention deficit hyperactivity disorder, cannabidiol, cannabis, marijuana, thc

## Abstract

Cannabis is frequently used by people who self-medicate for the signs of mental health conditions. Attention-deficit/hyperactivity disorder (ADHD), a neurodevelopmental illness, has been linked to increased cannabis use. However, compared to other mental disorders, cannabis use by people with ADHD has received much less research. The main goal of this systematic review was to understand the nature of the relationships between cannabis use and ADHD symptoms. We used Preferred Reporting Items for Systematic Reviews and Meta-Analyses (PRISMA) guidelines to conduct the systematic review. We included papers published within the previous ten years from online searches on PubMed, PubMed Central (PMC), Google Scholar, and ScienceDirect until January 1st, 2023. The inclusion-exclusion criteria led to the initial selection of 136 studies. We selected twenty research articles after screening and assessing them using quality assessment techniques. These articles included two non-randomized control trials, one cross-sectional study, one meta-analysis, and sixteen observational cohorts. It can be advantageous for people with ADHD and their medical professionals to understand better how ADHD patients use cannabis and its potential risks and advantages on cannabis use disorder, ADHD symptoms, and executive dysfunction. This article further emphasizes the necessity of thorough research to comprehend cannabis use in ADHD patients.

## Introduction and background

Attention-deficit hyperactivity disorder (ADHD) is a prevalent psychiatric disorder among children, with some cases persisting into adulthood. According to the Diagnostic and statistical manual of mental disorders (5th ed.) [DSM-V], six or more symptoms of inattention (such as making careless mistakes) and/or hyperactivity (such as restlessness) in children up to age 16 indicate ADHD. Adolescents 17 years and older and adults, however, must exhibit five or more symptoms [1]. According to studies, 2-12% of school-aged children have ADHD symptoms, and 60% of them have these symptoms as adults [2]. Children and adolescents with ADHD symptoms also have worse mood dysregulation and poor social and academic functioning [1].

About 90% of ADHD patients also have co-occurring mental problems as frequent comorbidity. According to reports, 15% of the general population and up to 50% of adults with ADHD use drugs or alcohol regularly [2]. Moreover, ADHD has been identified in about 23% of people with substance use disorders (SUD) [3]. After tobacco and alcohol, cannabis misuse is the most frequent of them [2]. Several processes might be at play in the elevated risk of SUD in ADHD. Instead of being causal, the relationships may be due to overlapping genetic and environmental variables that increase the chance of marijuana use disorder and attention deficit hyperactivity disorder (ADHD) [4]. Neurobiological theories claim that an imbalance in dopaminergic neurotransmission is the root cause of both diseases [5]. The self-medication hypothesis, which claims that people with ADHD may try various illegal substances and find that some help them with problematic emotional or affective states, is the reason for the association between drug use andthis theory [5].

Cannabis is one of the most widely used illicit narcotics globally, with significant usage rates in youth and early adulthood [6]. Cannabis (sometimes known as marijuana) is made from the dried leaves and flower seeds of the Cannabis sativa plant [6]. Cannabis usage, whether short- or long-term, has several impacts on the immunological, respiratory, cardiovascular, and central nervous systems [2], including diminished attention, reduced working memory, and diminished executive functioning [7]. Compared to children without ADHD, individuals with ADHD had nearly three times the likelihood of ever using marijuana and 1.5 times the likelihood of developing a cannabis dependence [6]. Medical cannabis (MC) is becoming more widely acknowledged as a potential alternative therapy for adult ADHD [8]. However, according to Hergenrather et al., there is still controversy in the use of MC for ADHD [8].

Stringent laws restricted cannabis usage in the past. Nevertheless, the transition is accelerating since various countries and nations have loosened or are considering loosening rules against its use and manufacture, making those with acute conditions like ADHD more susceptible to suffering the consequences. Given the reasons mentioned above, we conducted a thorough systematic review to determine the nature of the connection between cannabis and ADHD.

## Review

Methods 

We used the Preferred Reporting Items for Systematic Reviews and Meta-Analyses (PRISMA) 2020 criteria to plan and present the results of this systematic review.

*Search Strategy*

We performed in-depth searches in ScienceDirect, PubMed (MEDLINE), PubMed Central, and Google Scholar. To find all possibly pertinent papers discussing the connection between cannabis and ADHD, we employed the right keywords and Medical Subject Heading (MeSH) terminology. Attention deficit hyperactivity disorder, ADHD, attention deficit disorder with hyperactivity, and cannabis, marijuana, cannabidiol, and THC, were among the keywords utilized. We combined the keywords and MeSH phrases using the Boolean approach to create a constant search over multiple databases. After removing duplicates, we carefully examined our data to ensure all potentially relevant items were present. We next reviewed the titles and abstracts of the obtained records and further evaluated their relevance. After that, two researchers independently assessed full-text papers against the inclusion and exclusion criteria, with a third researcher acting as a tiebreaker.

Inclusion and Exclusion Criteria

We limited our search to records published online between January 2012 and January 2023 that were released in English and were freely accessible as complete texts. Meta-analyses, systematic reviews, literature reviews, randomized and non-randomized clinical trials, and observational prospective and retrospective cohorts were the only research we considered. To be included, studies needed to have participants who were adolescents or young adults (age 25) or later adults (>25), compare individuals with ADHD or sub-threshold ADHD who used cannabis or synthetic cannabinoids to those who did not and measure any neurodevelopmental outcome (defined broadly to include neuroimaging, symptoms, and functioning). Table 1 shows the search strategy and inclusion and exclusion criteria for the articles used in our systematic review.

**Table 1:** A summary of search strategies used in PubMed, PubMed Central, Google Scholar, and ScienceDirect.

Database	Keywords	Search strategy	Filters used	Results
PubMed	attention deficit hyperactivity disorder, attention deficit disorder with hyperactivity, marijuana, cannabidiol, thc	((("attention deficit hyperactivity disorder"[All Fields] OR ("attention deficit disorder with hyperactivity"[MeSH Terms] OR ("attention"[All Fields] AND "deficit"[All Fields] AND "disorder"[All Fields] AND "hyperactivity"[All Fields]) OR "attention deficit disorder with hyperactivity"[All Fields] OR "adhd"[All Fields])) AND ("attention deficit disorder with hyperactivity/cerebrospinal fluid"[MeSH Major Topic] OR "attention deficit disorder with hyperactivity/chemically induced"[MeSH Major Topic] OR "attention deficit disorder with hyperactivity/epidemiology"[MeSH Major Topic] OR "attention deficit disorder with hyperactivity/etiology"[MeSHMajor Topic] OR "attention deficit disorder with hyperactivity/genetics"[MeSH Major Topic] OR "attention deficit disorder with hyperactivity/metabolism"[MeSH Major Topic] OR "attention deficit disorder with hyperactivity/psychology"[MeSH Major Topic])) OR "attention deficit hyperactivity disorder"[All Fields]) AND ("2013/01/10 00:00":"3000/01/01 05:00"[Date - Publication] AND "loattrfree full text"[Filter] AND "humans"[MeSHTerms] AND "english"[Language]) AND (((("cannabis"[MeSH Terms] OR "cannabis"[All Fields] OR "cannabi"[All Fields] OR "cannabis s"[All Fields]) AND ("cannabis/adverse effects"[MeSH Major Topic] OR "cannabis/drug effects"[MeSH Major Topic] OR "cannabis/genetics"[MeSH Major Topic] OR "cannabis/physiology"[MeSH Major Topic] OR "cannabis/toxicity"[MeSH Major Topic])) OR ("cannabis"[MeSH Terms] OR "cannabis"[All Fields] OR "marijuana"[All Fields] OR "marijuana s"[All Fields]) OR ("cannabidiol"[MeSH Terms] OR "cannabidiol"[All Fields] OR "cannabidiolic"[All Fields]) OR ("dronabinol"[MeSH Terms] OR "dronabinol"[All Fields] OR "thc"[All Fields])) AND ("2013/01/10 00:00":"3000/01/01 05:00"[Date - Publication] AND "loattrfree full text"[Filter] AND "humans"[MeSHTerms] AND "english"[Language]))	Published between 2012-2023. Full-text articles, Human studies only, English language and papers translated to English.	59
PubMed Central	Adhd and cannabis		Articles published in last ten years.	12
Google Scholar	Adhd and cannabis		Articles published between 2012 and 2023.	29
Science Direct	Adhd and cannabis		Articles published between 2012 and 2023, review articles, and research articles in neuroscience, psychology, pharmacology, toxicology, and biochemistry, all journals except those in French.	36

Study Quality Appraisal

We evaluated every study's potential for bias. We assessed clinical studies using the updated Cochrane risk of bias 2 (RoB 2) algorithm. The Newcastle-Ottawa Scale (NOS) was used to evaluate cohort studies. We evaluated systematic reviews and meta-analyses using the Assessment of Multiple Systematic Reviews 2 (AMSTAR 2) tool. We examined each randomized controlled trial for potential biases based on five risks of bias using the most recent Cochrane RoB 2 methodology. We evaluated the quality of the cross-sectional study using the AXIS tool. Each bias risk was rated low, high, or moderate. Additionally, we specified whether there was a low, high, or moderate risk of bias overall. Table 2-5 shows the results of New Castle Ottawa Scale, AMSTAR 2 checklist, Cochrane Risk Bias tool, and AXIS tool respectively. 

**Table 2:** Summary of the Newcastle-Ottawa Scale for cohort studies. Key: 1, the exposed group represents the general population; 2, the non-exposed group is selected from the same population as the exposed group; 3, appropriate documentation of outcome; 4, absence of study outcome at the beginning of the cohort; 5, controlled study characteristics allow for comparison of the exposed group to non-exposed group; 6, blinded assessment of results; 7, adequate length of follow-up period; 8, complete or nearly complete follow-up of participants; NOS, Newcastle-Ottawa Scale; AHRQ, Agency for Healthcare Research and Quality; Y, yes; PY, partial yes.

First author (year)	Selected population (/4)1234	Comparison (/2)5	Results (/3)6	Final NOS (/9)	AHRQ standards
Tamm et al. [9]	YYYY	PY	YYY	8	Good quality
Harty et al. [10]	YYYY	PY	YYY	8	Good quality
Lee et al. [6]	YYYY	PY	YYY	8	Good quality
Lisdhal et al. [11]	YYYY	PY	YYY	8	Good quality
Aksoy et al. [2]	YYNY	PY	YYY	7	Good quality
Wallace et al. [7]	YYYY	PY	YYY	8	Good quality
Kolla et al. [12]	YYYY	PY	YYY	8	Good quality
Elkins et al. [4]	YYYY	PY	YYY	8	Good quality
Coetzee et al. [5]	YYYY	PY	YYY	8	Good quality
Tchuente et al. [13]	YYYY	PY	YYY	8	Good quality
Ly et al. [14]	YYYY	PY	YYY	8	Good quality
Patel et al. [15]	YYYY	PY	YYY	8	Good quality
Jean et al. [16]	YYYY	PY	YYY	8	Good quality
Hergenrather et al. [8]	YYYY	PY	YYY	8	Good quality
Kelly et al. [17]	YYYY	PY	YYY	8	Good quality
Stevens et al. [1]	YYYY	PY	YYY	8	Good quality

**Table 3:** Summary of the Assessment of Multiple Systematic Reviews 2 (AMSTAR 2) tool. Key: RoB, risk of bias; Y, yes; PY, probably yes; N, no; NA, non-applicable

First author (Year)	(1)PICO framework included	(2) Pre-defined methods and research proposal	(3)Design of the study outlined	(4)Thorough literature search	(5)Selection of studies by two individuals	(6)Extraction of data by two individuals	(7)Record and reasons for reports excluded	(8)Detailed description of included studies	(9) Adequate RoB procedure followed	(10)Disclosure of funding sources	(11)Appropriate statistical analysis	(12) Effect of RoB of primary studies on metanalysis	(13)RoB considered in primary studies	(14)Investigation of heterogenicity	(15)Small study bias	(16) Potential conflicts reported	Total score(/16)	Final quality appraisal of the review
Cawkwell et al. [18]	Y	Y	Y	Y	Y	Y	Y	Y	NA	Y	NA	NA	Y	NA	Y	N	15	High

**Table 4:** Assessment of clinical trials using the revised Cochrane risk of bias 2 tool. Key: RoB, risk of bias; LR, low risk; MR, moderate risk; HR, high risk.

First author (year)	Random allocation	Intervention non- adherence	Incomplete results	Inadequate assessment of the outcomes	Selective reporting	Final RoB judgment
Artigas et al. [19]	LR	LR	LR	LR	LR	LR
Chauchard et al. [20]	LR	LR	LR	LR	LR	LR

**Table 5:** Assessment of cross-sectional studies using the AXIS tool. Key: RoB, risk of bias; Y, yes; PY, probably yes; N, no; NA, non-applicable.

First author, year	Study design	Justified sample size	Appropriate population base	Representative population	Appropriate measurements	Correctly usage of instruments	Discussion/ conclusion	Ethical approval	Total points (/8)
Notzon et al. [21]	Y	Y	Y	Y	Y	Y	Y	Y	8

Results

**Figure 1:**
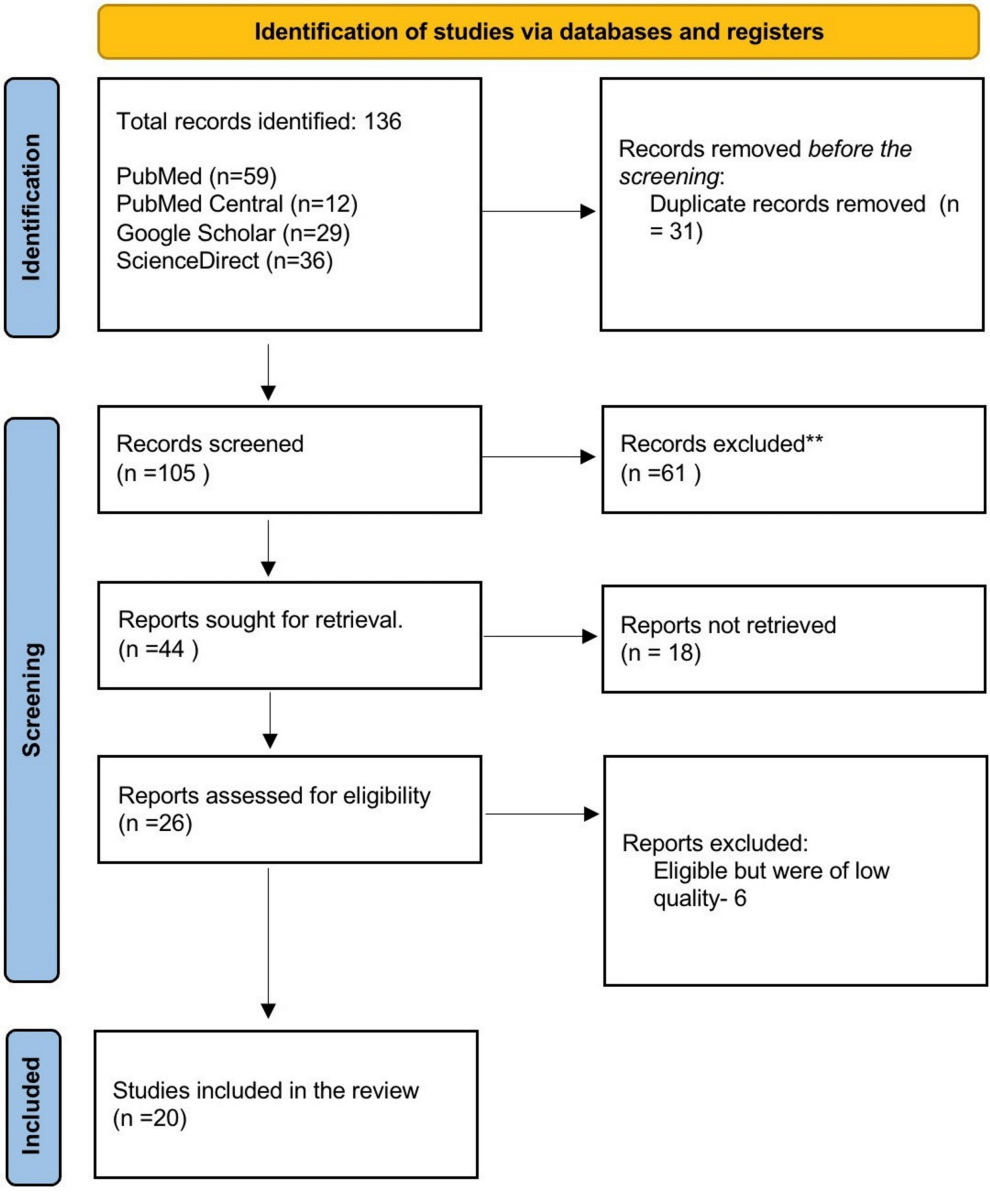
Preferred Reporting Items for Systematic Reviews and Meta-Analyses (PRISMA) flow diagram.

We identified 136 records using the various search strategies across the databases. Of 136 records, 59 originated from PubMed (MEDLINE), 29 from Google Scholar, 36 from ScienceDirect, and 12 from PubMed Central. We employed no additional sources. We removed 31 duplicates manually before screening articles. Following a thorough review of the remaining 105 records for relevance based on their titles and abstracts, we eliminated 61 due to their lack of relevance to the study's subject, goals, and inclusion and exclusion criteria. Hence, 44 articles were sought for retrieval, and after checking for free full texts, we further removed eighteen reports. We assessed 26 reports for quality and removed six articles due to low quality. Therefore, we included 20 articles in this review. These included sixteen observational cohorts, one meta-analysis, two randomized controlled trials, and one cross-sectional study. Figure 1 shows a PRISMA flow diagram to represent search strategies across various databases. 

Discussion

ADHD in Adolescents and Young Adults

**Figure 2:**
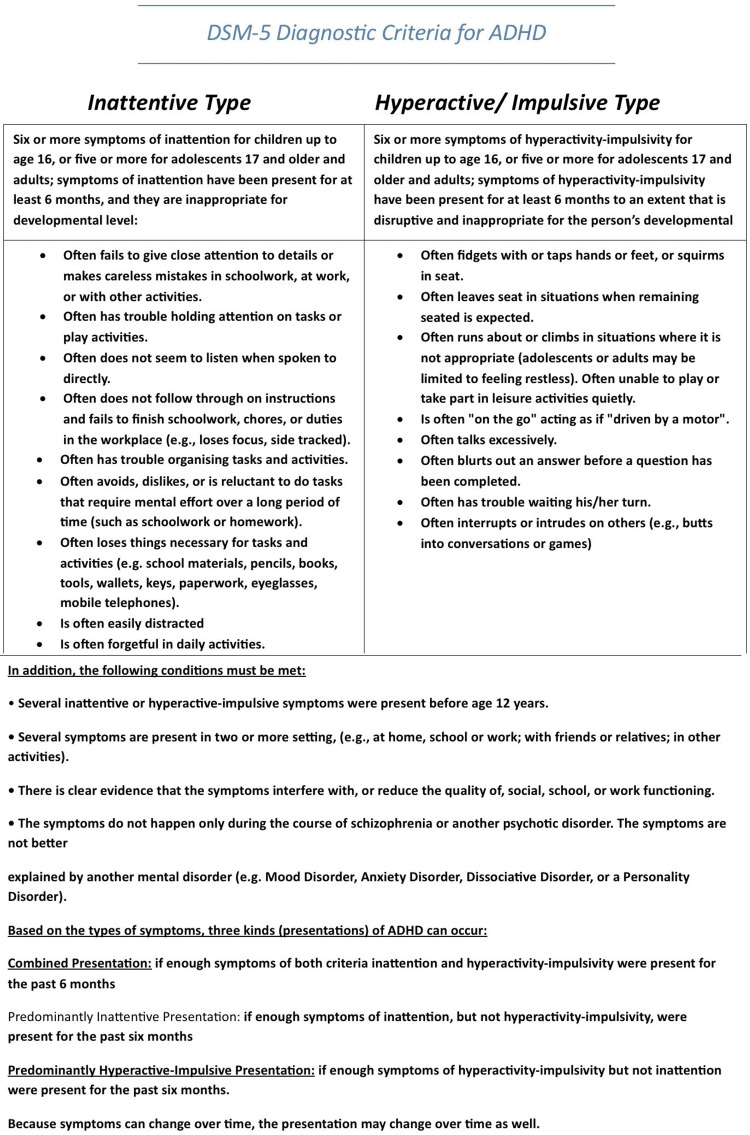
DSM-V criteria for diagnosis of ADHD in children, adolescents, and young adults. Key: DSM-V, Diagnostic and Statistical Manual of Mental Disorders-V; ADHD, Attention Deficit Hyperactivity Disorder.

According to several studies, most people with ADHD also have additional mental illnesses, with learning impairments, oppositional behavior, drug abuse, and anxiety being prevalent comorbidities [18]. Figure 2 describes the DSM-5 criteria for the diagnosis of ADHD in children and adults. Children with ADHD perform poorly in a variety of neuropsychological areas, including sustained attention, verbal and working memory, processing speed, response inhibition, reward processing, motivation, cognitive control, and impulsivity, as well as having mildly adverse effects on IQ in adolescence (though a potential independent impact of ADHD on IQ is controversial) [18]. Imaging studies have also revealed a wide range of variations in neurodevelopment in patients with ADHD, including changes in the structural organization of the brain as well as variations in the activation patterns of attentional networks and executive functions, including hypo-activation in the bilateral frontal, right parietal, right temporal, and bilateral putamen [18]. 

Cannabis Effects on the Human Brain

**Figure 3:**
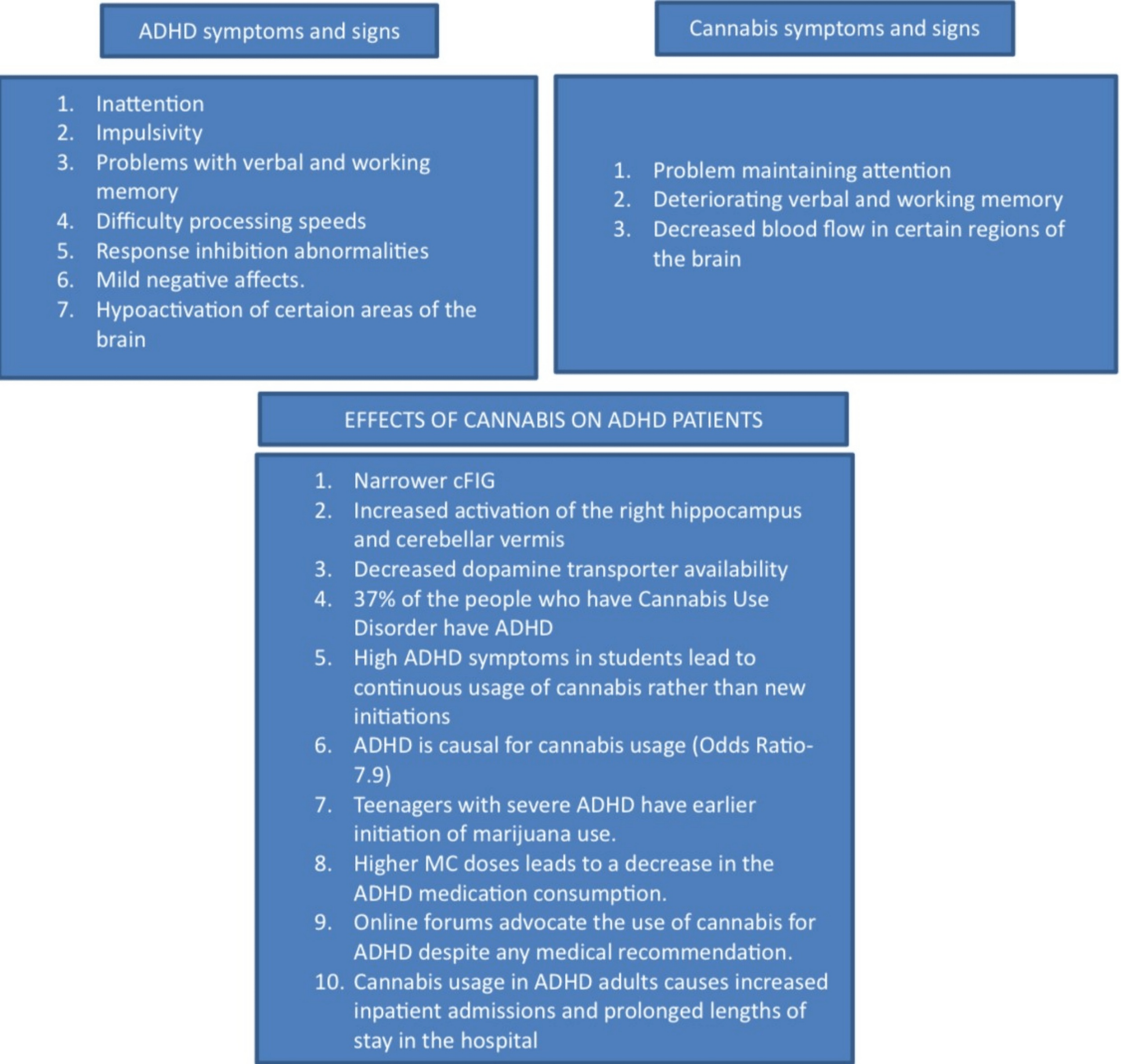
The relationship between Cannabis and ADHD has been briefly explained by this flowchart. Key: cFIG, cortical inferior frontal gyrus; ADHD, Attention Deficit Hyperactivity Disorder.

Regular cannabis use during adolescence is associated with diminished verbal and working memory and sustained attention [18]. Teenage cannabis usage may change the brain circuitry that controls motivation, impulsivity, processing rewards, reaction inhibition, and processing speed [18]. The amount of gray matter in the hippocampus and amygdala of young adults who use cannabis heavily is inversely connected with their use. There have been various cortical locations where less cerebral blood flow has been noticed [18]. Figure 3 is the flow diagram showing the relationship between Cannabis and ADHD.

Effects of Cannabis Intake on ADHD Patients

Structural changes in the brain: Numerous studies defined neurodevelopmental outcomes to include neuroimaging, cognitive testing, and questionnaire-based research to capture the developmental or functional effects of cannabis use on young people with ADHD. Rasmussen et al. found that cannabis use was associated with the diagnosis of ADHD in the right hippocampus and cerebellar vermis, with higher activation of these regions in cannabis-using controls during appropriate response inhibition [22]. Similar findings were reported by Newmann et al., who found that despite go/no-go performance, persistent ADHD symptoms and more frequent cannabis use were associated with a narrower caudal inferior frontal gyrus [9]. Similar results were reported by Colak et al. [23], who found synthetic cannabis users with ADHD had thinner cortical layers in the right precentral and postcentral gyri than controls. Lisdhal et al. did discover that early cannabis use at the age of onset in ADHD patients was associated with a thicker superior frontal and postcentral cortex in the right hemisphere [11]. However, Amen and Waugh observed that cannabis-using ADHD patients had bilateral temporal lobe hypoperfusion compared to those who did not [24]. Silva et al. [18] state that adolescents with ADHD and SUD (cannabis and cocaine) have lessened dopamine transporter availability. However, Kelly et al. [17], Tamm et al. [9], and Wallace et al. [7] found no evidence of a significant effect on the brain's structural organization and neurocognitive processes.

Substance use problems: In 90% of cases, ADHD coexists with another mental illness. 15% of the general population and up to 50% of adults with ADHD use alcohol or drugs, according to reports [2]. Between 34% and 46% of those who seek treatment for cannabis use disorders are thought to have ADHD [21]. The timing of the first use of cannabis by a student is not linked to ADHD symptoms, although high levels of ADHD symptoms might be a risk factor for students' continued use of cannabis, as well as their increasing usage of it [16]. The SUD/ADHD group had a higher rate of cannabis use [5]. In contrast to SUD-ADHD females, SUD+ADHD males, and SUD-ADHD males, SUD+ADHD females have expressly indicated a younger age of cannabis onset than SUD-ADHD females. They revealed that they also use cannabis for a more extended period than SUD-ADHD females [5]. According to Artigas et al. [19], there is a genetic link between ADHD and the likelihood of using cannabis and a causal link between ADHD and lifetime cannabis use.

Behavioral changes: Hyperactive and impulsive symptoms have been linked to problematic cannabis use in men [4,12]. However, only the inattentive symptomatology of women suggested cannabis-related problems [12]. In a different study, it was discovered that men with ADHD, but not women, had a strong correlation between increased marijuana use and an increase in symptoms of inattention [14]. Childhood ADHD is a predictor of the beginning and rate of marijuana use. These correlations are essentially explained by shared familial environment and genetics rather than causal factors [4].

Additionally, ADHD does not impact cannabis withdrawal in patients with cannabis dependence seeking therapy [20]. Sleep disturbance is a frequent justification for cannabis use among people who exhibit symptoms of ADHD [25]. Research demonstrates that people grow accustomed to cannabis' short-term sleep benefits, encouraging increased use and raising the risk of cannabis-related problems [26]. Comorbid CUD increases the likelihood that ADHD patients will require acute inpatient care and lengthens hospital stays, increasing care costs [15].

To overcome the harmful effects of ADHD: Adults with ADHD symptoms frequently cite low mood and negative affect as reasons for using cannabis to treat their symptoms [25]. This trend is also observable for cannabis coping reasons and cannabis difficulties in the general population [27], among veterans [28], and prospectively in an adult sample of regular cannabis users [29]. Adults with ADHD experience significant symptoms of social anxiety disorder (SAD) [30]. This comorbidity makes cannabis use disorders and other problems more likely in adults with symptoms of ADHD [31]. According to another study, consuming phytocannabinoids and terpenes in higher doses reduces the need for ADHD medication [8].

Perceived low risk: Despite the lack of clinical guidelines or thorough research to support these claims, online conversations indicate that marijuana is believed to be beneficial for ADHD. Cannabis veterans also believe that cannabis is less dangerous than other drugs or substances [32]. This type of internet data may impact how ADHD patients and their caregivers view cannabis use and treatment. Table 6 summarizes all the articles included in the systematic review, which shows the relationship between cannabis and ADHD in patients. 

**Table 6:** A list of the studies selected in this systematic review after the screening. fMRI: functional magnetic resonance imaging; MRI: Magnetic Resonance Imaging; SPECT: Single-photon emission computed tomography; DKEFS-CW: Delis–Kaplan Executive Function System Color Word Interference; WAIS: Wechsler Adult Intelligence Scale; WURS: Wender Utah Rating Scale; CAARS: Conners Adult ADHD Rating Scale; ASRS: ADHD Self-Report Scale; DIVA: Diagnostic-Interview for ADHD, GWAS: genome-wide association studies; ASRS-V1.1: Adult ADHD Self-Report Version 1.1; ASSIST: Alcohol, Smoking, and Substance Involvement Screening Test; DICA–R: Diagnostic Interview for Children and Adolescents-Revised; MJQQ: Marijuana Quit Questionnaire; ICD9–CM: International Classification of Diseases, 9th Revision, and Clinical Modification; PSQI: Pittsburgh Sleep Quality Index; GAD: General Anxiety Disorder; MC: Medical Cannabis; CUD: Cannabis Use Disorder; SUD + ADHD: Substance Use Disorder and Attention Deficit Hyperactivity Disorder.

Study	Number of participants	Methodology	Findings related to cannabis use in ADHD patients
Rasmussen at al. [22]	73	fMRI with go/no-go task	Activation of the right hippocampus and cerebellar vermis in cannabis using ADHD patients.
Newmann et al. [9]	114	fMRI with go/no-go task	Thinner caudal inferior frontal gyrus is present in cannabis-using ADHD patients.
Lisdahl et al. [11]	75	Structural MRI	Larger left nucleus accumbens and thicker right superior frontal and postcentral gyri in patients with earlier cannabis use.
Colak et al. [23]	41	Structural MRI	Thinner cortical layers were found in the right precentral and postcentral gyri in cannabis-using ADHD patients.
Amen & Waugh et al. [24]	40	SPECT	Contrary to individuals with ADHD who did not use cannabis, those with ADHD who did show bilateral temporal lobe hypoperfusion.
Silva et al. [18]	62	SPECT	Adolescents with ADHD and SUD (cannabis and cocaine) have less dopamine transporter availability.
Kelly et al. [17]	75	fMRI	No significant structural changes in ADHD patients using cannabis.
Tamm et al. [9]	128	Hopkins Verbal Learning TaskGo/no-go tasklowa Gambling Task DKEFS-CWI TestTrail-making task	No significant effects of cannabis are present in ADHD patients.
Wallace et al. [7]	72	Ruff 2&7 Conners Continuous Performance Test-II , WAIS III Letter Number Sequencing DKEFS-CWI	Cannabis use in ADHD patients had no significant impact on attention.
Notzon et al. [10]	99	WURS, CAARS, ASRS	34% and 46% of individuals seeking treatment for cannabis use problems have ADHD.
Jean et al. [16]	4270	ASRS	High ADHD symptoms in students could lead to continued cannabis use rather than new initiations.
Coetzee et al. [5]	185	DIVA 2.0	The SUD+ADHD group showed increased cannabis consumption.
Artigas et al. [19]	50,799	GWAS of ADHD using Mendelian randomization	ADHD is causal for lifetime cannabis use, with an odds ratio of 7.9 for cannabis use in individuals with ADHD compared to individuals without ADHD.
Kolla et al. [12]	5,080	ASRS-V1.1 and ASSIST	Inattentive symptomatology predicted problems with cannabis only in women.
Christine Ly and Jean-G. Gehricke [14]	76	Assessment of Hyperactivity and Attention drug use survey Pittsburgh Sleep Quality Index	Moderate to strong correlations were found between marijuana use and inattentive symptoms in men and marijuana use and decreased sleep quality in women.
Elkins et al. [4]	3,762	DICA–R; Reich, 2000 Substance Abuse Module	Teenagers who had more severe childhood ADHD were more likely to start drinking and using marijuana younger, use it frequently or heavily, and have symptoms.
Chauchard et al. [20]	23	MJQQ	ADHD does not influence cannabis withdrawal symptoms.
Patel et al. [15]	11,232	ICD9–CM codes.	Comorbid CUD in patients with ADHD increases the risk of acute inpatient care and prolongs the inpatient stay.
Hergenratger et al. [8]	3,218	Qualtrics® (version 12018; Provo, UT, USA) , ASRS-v1.1 ADHD rating scale, PSQI, GAD-7 scale	Higher-dose consumption of MC components is associated with ADHD medication reduction.
Mitchell et al. [25]	268 forum threads	Qualitative rating- three raters (Cohen’s kappa = 0.74)	There has been a rise in online forum discussions supporting cannabis use as a treatment for ADHD, despite the absence of any clinical advice or thorough research to back up these claims.

Limitations

Several of these studies [9], [14], and [19] only consider the diagnosis of childhood ADHD instead of adult ADHD symptoms. Additionally, previous studies have largely ignored the possibility of gender differences in cannabis consumption habits or problems with adult ADHD symptoms. The effectiveness of ADHD medications, the side effects of those treatments, or the effects of cannabis on the various ADHD symptoms have not been thoroughly investigated in earlier research. Except for a few studies [9], previous research has largely ignored the potential effects of cannabis use on executive dysfunction linked to ADHD.

## Conclusions

This systematic review sheds new light on the cannabis use patterns associated with ADHD symptoms, the perceived effects of cannabis on specific core symptoms of ADHD, and the potential moderating effects of cannabis use on executive functioning deficits related to ADHD. The significant findings, in particular, indicate a relationship between the frequency of cannabis use and the severity of ADHD symptoms, as well as a slightly increased risk of CUD. In conclusion, research on the effects of concurrent cannabis use in adolescents and young adults with ADHD is currently lacking. There is a need to study the detailed effects of ADHD medications and smoking cannabis on ADHD symptoms. Preliminary studies reveal abnormalities in perfusion, altered brain architecture, and altered density of dopamine transporters. The evidence to date is inconclusive regarding whether cannabis use has an addictive effect or interactions, whether beneficial or detrimental. The evidence base at this time is relatively small, so more study is needed. Understanding the neurodevelopmental impact of cannabis use is essential for both clinical and societal reasons, especially in populations like children with ADHD, who may be especially vulnerable to them, given the legalization's increased accessibility and use of cannabis and the potency of cannabis formulations.
