# Correction: Evaluation of Efficacy of Cannabis Use in Patients With Attention Deficit Hyperactivity Disorder: A Systematic Review

**DOI:** 10.7759/cureus.c313

**Published:** 2025-09-23

**Authors:** Divyanshu Dhamija, Adedamola O Bello, Asma A Khan, Sai Dheeraj Gutlapalli, Mehvish Sohail, Priyansh A Patel, Sidharth Midha, Surmai Shukla, Lubna Mohammed

**Affiliations:** 1 Internal Medicine, California Institute of Behavioral Neurosciences & Psychology, Fairfield, USA; 2 Psychiatry, St. Martinus University, Pontiac, USA; 3 Internal Medicine, Richmond University Medical Center, Staten Island, USA; 4 Internal Medicine, Medical College Baroda, Vadodara, IND; 5 Radiology, Bharati Vidyapeeth, Pune, IND; 6 Internal Medicine, Qingdao University College of Medical Science, Qingdao, CHN

This article has been corrected to fix a typo in the paragraph immediately preceding the PRISMA diagram. The number of articles excluded due to a lack of free full texts was erroneously stated to be "eight" and has been corrected to "eighteen". The PRISMA diagram is correct and remains unchanged.

