# Correction: Evaluation of Efficacy of Cannabis Use in Patients With Attention Deficit Hyperactivity Disorder: A Systematic Review

**DOI:** 10.7759/cureus.c378

**Published:** 2025-11-14

**Authors:** Divyanshu Dhamija, Adedamola O Bello, Asma A Khan, Sai Dheeraj Gutlapalli, Mehvish Sohail, Priyansh A Patel, Sidharth Midha, Surmai Shukla, Lubna Mohammed

**Affiliations:** 1 Internal Medicine, California Institute of Behavioral Neurosciences & Psychology, Fairfield, USA; 2 Psychiatry, St. Martinus University, Pontiac, USA; 3 Internal Medicine, Richmond University Medical Center, Staten Island, USA; 4 Internal Medicine, Medical College Baroda, Vadodara, IND; 5 Radiology, Bharati Vidyapeeth, Pune, IND; 6 Internal Medicine, Qingdao University College of Medical Science, Qingdao, CHN

This article has been corrected due to errors found within the reference list. Specifically, reference #3 has been added, reference 6 has been replaced, and reference 24 has been removed. As a result of these changes, references 3-24 have been renumbered and the text in the introduction section has been rearranged to ensure that the first instance of each reference appears in chronological order.

